# A patient-centred web-based adverse drug reaction reporting system identifies not yet labelled potential safety issues

**DOI:** 10.1007/s00228-021-03134-9

**Published:** 2021-06-18

**Authors:** J. Hasford, F. Bruchmann, M. Lutz, P. Thürmann, S. Schmiedl

**Affiliations:** 1 Institute for Medical Information Processing, Biometry, and, Epidemiology, University of Munich, Munich, Germany; 2 MEDIKURA Digital Health GmbH, Munich, Germany; 3 Department of Clinical Pharmacology, School of Medicine, Faculty of Health, Witten/Herdecke University, Witten, Germany; 4 Philipp Klee-Institute for Clinical Pharmacology, Helios University Hospital Wuppertal, Wuppertal, Germany

**Keywords:** Drug safety, Adverse drug reaction, ADR reporting by patients, SPC, Non-labelled ADR

## Abstract

**Purpose:**

Reporting of adverse drug reactions (ADRs) by patients is essential for a comprehensive risk–benefit evaluation of drugs after marketing, but only few data are available regarding patient-centred web-based ADR reporting systems. Hence, we aimed to analyze ADRs reported by patients with a particular emphasis on novel drugs and serious ADRs not yet labelled in the respective summary of product characteristics (SPC).

**Methods:**

All ADR reports received by a web-based, patient-centred platform (www.nebenwirkungen.de) between April 1, 2019, and September 1, 2020, were descriptively analyzed. ADRs and drugs were coded automatically according to MedDRA and ATC classification system. SPC labelling of reported ADRs for novel drugs marketed since 2015 was checked manually.

**Results:**

In total, 13,515 patient reports including 29,529 ADRs were received during the study period (serious ADRs [SADRs] *n* = 1,318; 4.5%). Women were affected in more than two-thirds of ADR reports. The most common patient-reported ADRs were nausea, dizziness and headache, whereas arrhythmia, intestinal obstruction and erectile dysfunction were the most frequent SADRs. Ciprofloxacin, levothyroxine and venlafaxine were the compounds most frequently suspected for causing both ADRs and SADRs. Regarding novel compounds, 289 reports including 739 ADRs were received (mainly fatigue, headache and myalgia). Three hundred thirty-one (44.8%) out of those ADRs were not yet labelled in the respective SPC, whereof twelve were SADRs.

**Conclusion:**

The majority of patient-reported ADRs were non-serious. However, a relevant number of non-labelled even serious ADRs was reported for novel compounds by patients. Despite well-known limitations of patient-reported ADRs, this web-based ADR reporting system contributes to the identification of new ADRs and thus can help to improve patients’ safety complementing other pharmacovigilance instruments.

**Supplementary Information:**

The online version contains supplementary material available at 10.1007/s00228-021-03134-9.

## Introduction

Since the seminal paper by Egberts et al. [1] in 1996 on adverse drug reactions (ADRs) showed that reports by patients can identify new ADRs faster than physicians’ reports, it became well accepted that patient-centred ADR reporting systems are an essential part of a comprehensive and state-of-the-art pharmacovigilance system. Thus, since 2011, the EU member states are asked to encourage patients to report ADRs [2]. In 2019, about 16.5% of all ADR reports to the EudraVigilance database were received from 122,073 patients [3]. Given that there were about 500 million people living in the EU and almost half of them used prescription medicines and about 20% of them use non-prescription drugs [4], it is obvious that these consumer reports were only the tip of the iceberg. All EU member states accept suspected AE/ADR reports from patients and consumers, but there is very little promotion and encouragement to do so seen in most of the member states. This negligence is in contrast to studies performed by Blenkinsopp et al., Watson et al. and Rolfes et al. that emphasized the value of patient reports and confirmed that the data quality is comparable to physician reports [5–7]. Given that e-health tools for pharmacovigilance have been available for 20 years, it seems that they are still not widely used in Europe [8].

As the web and smart phones have become meanwhile almost indispensable tools to master everyday’s life, we considered it worth to develop and evaluate a web-based platform for the collection and processing of patients’ reports about suspected AEs/ADRs. In 2018, a spin-off of the Technical University of Munich (TUM) started a new web-based patient-centred AE/ADR reporting system with the aim to improve the current situation by developing a multi-directed IT infrastructure solution for the real-time communication of suspected ADRs between all key stakeholders in the healthcare system, i.e. patients, healthcare professionals (HCP), manufacturers/marketing authorization holders (MAH), regulators and other healthcare providers.

As there are now more than 13,000 patient reports available, we aim to present the first results. We focused our analyses on the type of users of the system, the most frequently reported suspected AEs/ADRs and alleged drugs, and whether the system is useful for the evaluation of the safety profile of newly approved drugs and for the identification of potential ADRs not yet labelled in the respective Summary of Product Characteristics (SPC).

## Methods

Patients use the web-based platform via direct access to the web domain www.nebenwirkungen.de or through a large affiliated partner network to report suspected adverse drug reactions (ADRs). Developed as a customizable reporting solution, digital health applications make use of the proprietary infrastructure to extend their service offerings to all patients that experience ADRs and have access to the internet. The report can be completed by filling in the suspected adverse drug reactions, their duration, the administered drugs including their start and end date, dosage as well as the suspected drug names, pre-existing conditions, sex, pregnancy, weight, height, and further free text. Each report receives an internal report ID, and information, such as the ADR and administered drugs are automatically coded according to the most recent MedDRA and ATC classification. The technical feature of the system only permits suspected ADR reports with a plausible temporal association between the reaction and the suspected causative drug, i.e. the onset date of the reaction must not be prior to the start date of the suspected drug. The reporter is asked to enter the suspected drug at the first position, indicated as “primary suspicious”, followed by the non-suspicious concomitant medications (if there are any). The reporter also has the opportunity to indicate more than one suspected drug. Furthermore, an automatic seriousness check is completed based on the Important Medical Events (IME) list of the European Medical Agency (EMA) [9]. After an internal quality assurance process performed by a pharmacologically experienced team, the report is automatically assigned to the marketing authorization holder (MAH) of the suspected drug. The quality assurance includes a duplicate and plausibility check of the data provided. In very rare cases of an obviously wrong selection of the suspected drug by the reporter, the assignment is changed manually and the report directed to another MAH based on medical judgement considering the pharmacological profile of the indicated medication. The MAH then processes the report in accordance with the international pharmacovigilance guidelines valid in the EU, may initiate via the platform follow-ups of patients and healthcare professionals where necessary, and after completion, transmits the report to the EudraVigilance database. One of the particularities of the system is the multi-directed digital communication between all parties involved, such as that MAHs can ask follow-up questions to the patients via the platform to get further information for a better case assessment. Reports are transmitted to the manufacturer in a pseudonymous way, i.e. the personal identity of the patient remains protected and is only known to the HCPs involved. For this purpose, the personal data of the reporting patients are stored separately from the medical data to ensure the highest possible level of data protection.

We use the term adverse drug reaction (ADR) as the EMA stated that “for regulatory reporting purposes, as detailed in ICH-E2D (…), if an event is spontaneously reported, even if the relationship is unknown or unstated, it meets the definition of an adverse drug reaction” [9]. In this study, recently approved drugs were defined as compounds with a market access in Germany since January 1, 2015. A descriptive analysis was performed in which metric variables were reported as mean ± standard deviation or as median (first quartile–third quartile). Categorical variables were presented as frequency and percentage. Analyses were conducted using SAS statistical software package, version 9.2 (SAS Institute Inc., Cary, NC, USA) or SPSS (Statistical Package for Social Science, IBM®, USA).

## Results

### Demographics

Between April 1, 2019, and September 1, 2020, *n* = 13,515 reports were received. More than 30% of MAHs use the option to contact the reporter, with a 55% response rate by patients. More than two-thirds of the reports with documented age and sex (*n* = 13,116) came from women (e-Table 1). For women, the highest number of reports was received by the age group “21 to 30 years”, whereas in men, most frequent reports were made by the age groups “71 to 80 years” (e-Table 1). The mean age was 44.5 ± 18.0 years for women and 55.6 ± 20.6 for men.

### Suspected compounds in patient-reported ADRs and serious ADRs (SADRs)

Regarding the reports received within the study period (*n* = 13,515), the mean number of drugs taken was 1.4 ± 1.1 (range: 1 to 26), whereas the mean number of ADR-suspected drugs was 1.0 ± 0.3 (range: 1 to 9). The mean number of suspected ADRs per report was 2.2 ± 1.9 (range: 1 to 33). Out of all *n* = 29,529 patient-reported ADRs, *n* = 1,318 (4.5%) fulfilled the IME criteria for “serious”.

With regard to the twenty most frequently suspected compounds, 13 compounds including the top 3 compounds were listed in both the ADR and the SADR groups (e-Table 2). However, five contraceptive compounds were part of the ADR group but only one contraceptive was listed in the SADR group. On the other hand, two beta-1-adrenoceptor antagonists and two gadolinium-containing contrast media agents were part of the most prevalent SADR group but not listed in the ADR group.

Comparing the most frequently patient-reported ADRs with SADRs (top 20 group), different reactions were found (e-Table 3). The most common alleged ADRs were nausea, dizziness, headache, fatigue, and diarrhea, whereas the most common serious ADRs were arrhythmia, intestinal obstruction, erectile dysfunction, suicidal ideation, and circulatory collapse (e-Table 3).

For each of the ten most frequent patient-reported ADRs, the three most frequently suspected compounds (top three) are given in Table 1. Levothyroxine and venlafaxine were part of the top three most frequent causative compounds in seven and in five of the ten most frequent ADRs, respectively. The majority of reported ADRs have to be considered well known for the respective compounds. For some reported ADRs (e.g. levothyroxine-related weight increase), bias by indication might be an issue.

**Table 1:** Most frequently reported ADRs (number of reports, top ten, preferred terms according to MedDRA) related to the three most frequently suspected compounds

Adverse drug reactions	Number of reports	Suspected compound (top three)	ATC code	Number of reports
Nausea	1470	Levothyroxine	H03AA01	36
		Levonorgestrel and ethinylestradiol	G03AA07	32
		Venlafaxine	N06AX16	31
Dizziness	1416	Candesartan	C09CA06	42
		Venlafaxine	N06AX16	37
		Ciprofloxacin	J01MA02	34
		Methocarbamol	M03BA03	34
		Progesterone	G03DA04	34
Headache	1124	Levothyroxine	H03AA01	52
		Levonorgestrel and ethinylestradiol	G03AA07	26
		Ciprofloxacin	J01MA02	26
Fatigue	1114	Levothyroxine	H03AA01	45
		Ciprofloxacin	J01MA02	32
		Venlafaxine	N06AX16	28
Diarrhea	1015	Metformin	A10BA02	103
		Levothyroxine	H03AA01	33
		Amoxicillin and beta-lactamase inhibitor	J01CR02	32
Pruritus	601	Candesartan	C09CA06	14
		Levothyroxine	H03AA01	14
		Metamizole	N02BB02	14
Vomiting	479	Doxycycline	J01AA02	18
		Ibuprofen	M01AE01	13
		Tilidine	N02AX01	11
Rash	478	Amoxicillin	J01CA04	30
		Metamizole	N02BB02	26
		Amoxicillin and beta-lactamase inhibitor	J01CR02	14
Weight increased	477	Venlafaxine	N06AX16	43
		Levothyroxine	H03AA01	23
		Escitalopram	N06AB10	22
Hyperhidrosis	443	Venlafaxine	N06AX16	72
		Duloxetine	N06AX21	28
		Levothyroxine	H03AA01	28

Regarding the three most frequently suspected compounds for each of the ten most frequent SADRs (Table 2), some interesting results were found. For the most frequent SADR arrhythmia, levothyroxine is a well-known dose-dependent causative compound. For ciprofloxacin, somewhat benign tachycardia but also much more dangerous torsade de pointes arrhythmias are labelled adverse drug reactions. For several other patient-reported SADRs, suspected compounds are well-known causative agents (e.g. bisoprolol-related erectile dysfunction, citalopram-related suicidal ideation) but there are also some more or less unexpected SADR-drug pairs, e.g. levothyroxine- or pantoprazole-associated intestinal obstruction, and atorvastatin-related angina pectoris. However, bias by indication may play a role for some drug-SADR pairs (e.g., atorvastatin-related angina).

**Table 2:** Most frequently reported SADRs (number of reports, top ten, preferred terms according to MedDRA) related to the three most frequent suspected compounds

Serious adverse drug reaction	Number of reports	Suspected compound (top three)	ATC code	Number of reports
Arrhythmia	94	Levothyroxine	H03AA01	10
		Ciprofloxacin	J01MA02	9
		Metoprolol	C07AB02	7
Intestinal obstruction	82	Levothyroxine	H03AA01	5
		Oxycodone	N02AA05	4
		Pantoprazole	A02BC02	3
		Metformin	A10BA02	3
Erectile dysfunction	79	Ramipril	C09AA05	10
		Bisoprolol	C07AB07	6
		Amlodipine	C08CA01	3
		Pregabalin	N03AX16	3
		Citalopram	N06AB04	3
Suicidal ideation	64	Ciprofloxacin	J01MA02	6
		Citalopram	N06AB04	5
		Venlafaxine	N06AX16	5
Circulatory collapse	44	Cefuroxime	J01DC02	3
		Metamizole	N02BB02	3
		Bisacodyl	A06AB02	2
		Enoxaparin	B01AB05	2
		Sertraline	N06AB06	2
Syncope	43	Methocarbamol	M03BA03	3
		Metoprolol	C07AB02	2
		Tamsulosin	G04CA02	2
		Ciprofloxacin	J01MA02	2
		Ibuprofen	M01AE01	2
Hallucination	40	Levodopa and decarboxylase inhibitor	N04BA02	3
		Dihydrocodeine	N02AA08	2
		Tilidine	N02AX01	2
		Metamizole	N02BB02	2
		Mirtazapine	N06AX11	2
		Bupropion	N06AX12	2
		Venlafaxine	N06AX16	2
		Montelukast	R03DC03	2
Angina pectoris	34	Levothyroxine	H03AA01	3
		Metoprolol	C07AB02	2
		Amlodipine	C08CA01	2
		Atorvastatin	C10AA05	2
		Olodaterol and tiotropium bromide	R03AL06	2
Hemorrhage	33	Desogestrel	G03AC09	4
		Rivaroxaban	B01AF01	2
		Apixaban	B01AF02	2
		Misoprostol	G02AD06	2
		Progesterone	G03DA04	2
		Acetylsalicylic acid	N02BA01	2
Hematochezia	32	Acetylsalicylic acid	B01AC06	3
		Diclofenac	M01AB05	3
		Ustekinumab	L04AC05	2
		Ibuprofen	M01AE01	2
		Diphenhydramine	R06AA02	2

### Suspected compounds in patient-reported ADRs focusing on recently approved drugs

Considering ADR-suspected drugs approved for marketing in Germany since January 1, 2015, there were altogether 289 reports including 739 suspected ADRs. e-Table 4 shows the top 20 ADR-suspected drugs marketed since January 1, 2015. Edoxaban, a novel direct oral anticoagulant (DOAC), was the compound with the highest number of ADR reports. Novel oral antineoplastic compounds and monoclonal antibodies were the largest compound groups within the most prevalent compounds. Furthermore, a novel herpes zoster vaccination was suspected in 86 reports, i.e. the third most frequently alleged compound. The ten most frequent patient-reported ADRs reported for compounds marketed since January 1, 2015 were nausea, fatigue and headache (e-Table 5).

### Patient-reported ADRs/SADRs not yet labelled in the respective summary of product characteristics (SPC) for recently approved compounds

Altogether, 331 out of 739 (44.8%) patient-reported ADRs were not yet labelled in the respective SPC (Table 3). The most frequent ADRs not labelled for recently approved drugs were fatigue, headache and myalgia. For the novel and widely used DOAC compound edoxaban, several patient-reported ADRs were not yet listed (e.g. fatigue, dyspnoea, weight increase and abdominal pain upper).

**Table 3:** Most frequently reported ADRs (number of reports, top ten, preferred terms according to MedDRA, *n* > 1) related to novel compounds (marketed since January 01, 2015) not yet labelled in the respective SPC

Adverse drug reactions	Number of reports	Suspected compound	ATC Code	Number of reports
Fatigue	17	Evolocumab	C10AX13	4
		Vortioxetine	N06AX26	4
		Edoxaban	B01AF03	3
Headache	12	Dulaglutide	A10BJ05	3
		Evolocumab	C10AX13	3
		Alirocumab	C10AX14	2
		Baricitinib	L04AA37	2
Myalgia	11	Evolocumab	C10AX13	7
		Benralizumab	R03DX10	2
Dizziness	11	Dulaglutide	A10BJ05	6
		Evolocumab	C10AX13	2
Dyspnoea	9	Edoxaban	B01AF03	4
		Ixekizumab	L04AC13	2
Memory impairment	7	Evolocumab	C10AX13	3
Weight increased	6	Edoxaban	B01AF03	4
Abdominal pain upper	6	Edoxaban	B01AF03	2
Alopecia	6	Edoxaban	B01AF03	2
		Secukinumab	L04AC10	2
Pain in extremity	5*	Edoxaban	B01AF03	2
		Evolocumab	C10AX13	2
Visual impairment	5*	Edoxaban	B01AF03	2

^*^Due to the same number, both ADRs are shown

There were 12 patient-reported SADRs associated with novel compounds not yet labelled in the respective SPC (Table 4). Three SADRs (arrhythmia, respiratory disorder, asphyxia) were possibly related to the combination drug valsartan/sacubitril. Two SADRs were possibly related to secukinumab (intestinal perforation, Douglas’ abscess) and to Zoster, purified antigen (sudden hearing loss and syncope).

**Table 4:** SADRs (preferred term according to MedDRA) related to novel compounds (marketed since January 01, 2015) not yet labelled in the respective SPC

No	Serious adverse drug reaction	Suspected compound	ATC code
1	Arrhythmia	Valsartan and sacubitril	C09DX04
2	Respiratory disorder	Valsartan and sacubitril	C09DX04
3	Asphyxia	Valsartan and sacubitril	C09DX04
4	Sudden hearing loss	Zoster, purified antigen	J07BK03
5	Syncope	Zoster, purified antigen	J07BK03
6	Intestinal perforation	Secukinumab	L04AC10
7	Douglas’ abscess	Secukinumab	L04AC10
8	Circulatory collapse	Dulaglutide	A10BJ05
9	Anaphylactic shock	Ocrelizumab	L04AA36
10	Angina pectoris	Erenumab	N02CD01
11	Erectile dysfunction	Edoxaban	B01AF03
12	Drug addiction	Sofosbuvir and velpatasvir	J05AP55

## Discussion

The patient-centred adverse drug reaction reporting system (PARRS) was successfully implemented and made public, and within a short time received considerably many reports. Probably due to an easily understandable domain name (“Nebenwirkung” = ADR) and a user-friendly screen surface, patients felt addressed and were willing to report. Moreover, the data quality is higher as compared to other public reporting channels. Whereas an analysis of spontaneous reports submitted to the WHO VigiBase has revealed that 26% of the reports lacked data on age and 6% on sex [10], more than 97% of the PARRS reports contained all basic information, such as age and sex, respectively.

Interestingly, more than two-thirds of the reports were submitted from women. This can be explained at least in part by the fact that women use comparatively more drugs than men [4]. However, this gender discrepancy is in accordance with findings from global ADR patient reporting systems, where the majority of spontaneous reports come from health care professionals [11].

About 5% of all patient-reported adverse drug reactions were considered serious. Regarding ADR-suspected compounds marketed in Germany since 2015, 45% of patient-reported ADRs and twelve patient-reported SADRs were not yet labelled in the respective SPC.

Thus, it is obvious that PARRS may provide relevant new information about the safety profile of drugs extending existing pharmacovigilance systems focusing on healthcare professionals. Although it is difficult to assess causality in single cases, in particular when reported by consumers as essential information may be missing, PARRS allows to recontact the reporter and to ask for more detailed information. Moreover, via the reporter, also healthcare professionals can be contacted.

In three out of the twelve patient-reported SADR not yet labelled in the respective SPC, valsartan/sacubitril were considered as suspected compounds (arrhythmia, respiratory disorder, asphyxia). In the respective SPC, angioedema is listed as an adverse drug reaction potentially deteriorating ventilation. However, since abdominal complaints are also described as symptoms of angioedema [12], one should not consider the patient-reported airway-related ADRs (respiratory disorder, asphyxia) as labelled in the respective SPC by the term “angioedema”. The third patient-reported SADR for valsartan/sacubitril not yet labelled in the respective SPC was “arrhythmia”. Electrolyte disturbances (e.g. labelled hyper- or hypokalemia) may have led to such a serious consequence as arrhythmia.

For secukinumab, a novel compound to treat, i.e. psoriatic arthritis, “abdominal cramps and pain, diarrhea, weight loss, or blood in the stool (signs of bowel problems)” are documented gastrointestinal ADRs in the respective SPC. However, “intestinal perforation”, as reported by a patient in our study, is a life-threatening condition which has to be treated immediately and which cannot be considered as labelled in the respective SPC. From a clinical point of view, the SPC is the most important source for physicians to treat a patient appropriately and to allow a sufficient benefit-risk assessment in an individual patient. Hence, a regular update of the SPC is of outstanding importance to avoid preventable patient’s harm.

PARRS cases can, provided to EudraVigilance of the EMA and to VigiBase of the WHO Monitoring Center, even now support signal detection and verification. In the future, PARRS may also be used to apply signal detection methods to generate signals and to validate signals from other databases [13–15] too.

Meanwhile, most drug authorities in the EU accept online reporting and provide suitable report forms, but numbers of reports remain comparatively low. The German Authority for Medicinal Products and Devices, BfArM, received 4,832 reports directly from patients in 2019 of which 5.3% were classified as serious [16]. PARRS received almost fourfold more reports in the same time, whereas the proportion of serious cases remained similar. Hence, PAARS can be considered as complementary patient-centred ADR reporting system reaching additional patient populations. The multi-directional approach of PARRS connecting the patient, HCPs and MAHs in one platform to enable direct communication and to offer feedback may be a considerable advantage as the reporting to public authorities is rather unidirectional.

Although the number of reports received decreased slightly during the COVID-19 pandemic-induced lockdown in Germany from mid-March until the beginning of May 2020, it increased again to a level not achieved before COVID-19, although the number of physician visits had declined sharply and did not yet recover completely. Since the second lockdown starting 2 November, reporting numbers increased to 90 reports per day, a level not reached before (data not shown). Thus, PARRS enables safety reporting when the chances to communicate with a physician are limited. As it is not known how long COVID-19 will strain the healthcare system, web-based systems like PARRS may essentially help to compensate the losses due to reduced physician visits.

## Limitations

Since many years, the weaknesses of reporting single cases of alleged ADRs, in particular of patients, are well known. ADRs that mimic common diseases will be less often reported. Patients can only report AEs which they can perceive with their senses, but typically not the ones that need lab tests or imaging. Even if PARRS will reduce underreporting, there is no doubt that the reports collected do not allow to estimate absolute frequencies or incidences. As Rawlins stated already in 1994, causality assessment cannot be performed by patients [17]. Even the individual causality assessment by an expert in PARRS has limitations. For example, medical conditions will be incorrectly stated by some patients potentially impacting the assessment of alternative causes for the respective ADR. Interestingly, Rolfes et al. reported that the quality of patient reports compares well with healthcare professional reports [7]. In addition, due to the common lack of lab values, these data are also not part of the report. Furthermore, one can argue that a patient is not able to report certain diagnoses, e.g. angioedema correctly. However, in this study, a careful mapping of reported complaints to MedDRA was done by an expert team trained in pharmacovigilance.

The users of PARRS may not be a representative sample of the population, given that 71.6% of the reporters are female. However, 4.1% of all reports affected infants and children below up to the age of 10 years, and 15.0% elderly patients above 70 years. Both are typically underrepresented groups in clinical trials; thus, this source of information is highly valuable.

## Perspectives

Patient-reported outcomes get more and more accepted by the scientific community and drug regulators. Safety issues count certainly among the most important outcomes. As increasingly more new medicines get approved based on the results of phase II studies only, new approaches are definitely needed to optimize the assessment of the safety of these new drugs, when comparatively few patients have been exposed often for a short time only prior to the marketing authorization. The situation is similar for personalized medicines, and medical devices. The post-authorization safety studies (PASS) for medicinal products and the post-market clinical follow-up studies (PMCF) for medical devices prove that there is definitely a need to collect safety data in routine care to provide real-world safety evidence.

Thanks to the high level of flexibility of PARRS, this tool can be customized for different application areas, such as post-marketing studies, in real-world settings, clinical trials or as a healthcare app for everyday use. There is no doubt left that direct patient reporting plays a major role to the knowledge base about the safety profile of a new drug [13–15]. As even the elderly population gets increasingly familiar with IT solutions, the use of web-based systems will allow even this frequently neglected group to contribute to the safety knowledge base regarding their age group. The advantage of systems like PARRS is that it can be used to collect more information than just safety data, e.g. data on quality of life, and other outcomes like effectiveness measures that can be provided by the users. For a comprehensive assessment of the safety profile of medicinal products, various approaches are needed: database-based research, cohort and case–control studies, in certain situations randomized trials, and healthcare professional-focused reporting systems. But web-centred patient reporting systems represent an indispensable tool too. We definitely agree with a statement by Ignacio et al.: “Patient reporting adds new information, and perspective about ADRs in a way otherwise unavailable.” [14].

## Supplementary Information

Below is the link to the electronic supplementary material.

Supplementary file1 (DOCX 24 KB)

## Data Availability

Data archiving is not mandated but data will be made available on reasonable request.
