# Supplementary material for: A patient-centred web-based adverse drug reaction reporting system identifies not yet labelled potential safety issues
Source: Eur J Clin Pharmacol. 2021 Jun 18;77(11):1697–704. doi: 10.1007/s00228-021-03134-9 (PMC8212270; doi:10.1007/s00228-021-03134-9)
Supplement: Supplementary file 1 — Supplementary file1 (DOCX 24 KB) [file 228_2021_3134_MOESM1_ESM.docx]

**E-tables**

E-table 1: Number of ADR reports stratified by sex (male, female) and 10-years age groups

| age category | females (%) | males (%) | total (%) |
| --- | --- | --- | --- |
| 0-10 | 126 (1.3) | 126 (3.4) | 252 (1.9) |
| 11-20 | 593 (6.3) | 126 (3.4) | 719 (5.5) |
| 21-30 | 1,818 (19.4) | 309 (8.2) | 2,127 (16.2) |
| 31-40 | 1,670 (17.8) | 310 (8.3) | 1,980 (15.1) |
| 41-50 | 1,430 (15.3) | 411 (10.9) | 1,841 (14.0) |
| 51-60 | 1,806 (19.3) | 657 (17.5) | 2,463 (18.8) |
| 61-70 | 1,099 (11.7) | 779 (20.7) | 1,878 (14.3) |
| 71-80 | 646 (6.9) | 783 (20.8) | 1,429 (10.9) |
| 81-90 | 160 (1.7) | 234 (6.2) | 394 (3.0) |
| > 90 | 12 (0.1) | 21 (0.6) | 33 (0.3) |
| Total | 9,360 (100.0) | 3,756 (100.0) | 13,116 (100.0) |

E-table 2: Most frequently suspected compounds (prevalence ≥ 1%) for ADRs and SADRs

| Adverse drug reactions (n=29,529) | | | Serious adverse drug reactions (n=1,318) | | | |
| --- | --- | --- | --- | --- | --- | --- |
| compound | ATC Code | Number of ADRs (%) | compound | ATC Code | Number of SADRs (%) |  |
| ciprofloxacin | J01MA02 | 986 (3.3) | ciprofloxacin | J01MA02 | 67 (5.1) |  |
| levothyroxine | H03AA01 | 893 (3.0) | levothyroxine | H03AA01 | 35 (2.7) |  |
| venlafaxine | N06AX16 | 694 (2.4) | venlafaxine | N06AX16 | 33 (2.5) |  |
| candesartan | C09CA06 | 485 (1.6) | metoprolol | C07AB02 | 21 (1.6) |  |
| levonorgestrel and ethinylestradiol | G03AA07 | 450 (1.5) | ramipril | C09AA05 | 19 (1.4) |  |
| escitalopram | N06AB10 | 423 (1.4) | gadoteric acid | V08CA02 | 19 (1.4) |  |
| metformin | A10BA02 | 402 (1.4) | candesartan | C09CA06 | 18 (1.4) |  |
| citalopram | N06AB04 | 364 (1.2) | sertraline | N06AB06 | 18 (1.4) |  |
| ibuprofen | M01AE01 | 356 (1.2) | misoprostol | G02AD06 | 17 (1.3) |  |
| atorvastatin | C10AA05 | 354 (1.2) | ibuprofen | M01AE01 | 17 (1.3) |  |
| ramipril | C09AA05 | 349 (1.2) | metamizole | N02BB02 | 17 (1.3) |  |
| desogestrel | G03AC09 | 347 (1.2) | escitalopram | N06AB10 | 17 (1.3) |  |
| sertraline | N06AB06 | 335 (1.1) | citalopram | N06AB04 | 17 (1.3) |  |
| dienogest and ethinylestradiol | G03AA16 | 330 (1.1) | duloxetine | N06AX21 | 16 (1.2) |  |
| metamizole | N02BB02 | 330 (1.1) | acetylsalicylic acid | B01AC06 | 15 (1.1) |  |
| vaginal ring with progestogen and estrogen | G02BB01 | 318 (1.1) | atorvastatin | C10AA05 | 15 (1.1) |  |
| duloxetine | N06AX21 | 317 (1.1) | pregabalin | N03AX16 | 15 (1.1) |  |
| levofloxacin | J01MA12 | 305 (1.0) | pantoprazole* | A02BC02 | 14 (1.1) |  |
| plastic intrauterine device with progestogen | G02BA03 | 303 (1.0) | bisoprolol* | C07AB07 | 14 (1.1) |  |
| amoxicillin | J01CA04 | 277 | plastic intrauterine device with progestogen* | G02BA03 | 14 (1.1) |  |
|  |  |  | amitriptyline* | N06AA09 | 14 (1.1) |  |
|  |  |  | gadobutrol* | V08CA09 | 14 (1.1) |  |

*Due to the same number of SADRs, all five compounds are shown

E-table 3: Most frequently reported ADRs and SADRs (number of reports, top 20, Preferred Terms according to MedDRA)

| Adverse drug reactions | | Serious Adverse drug reactions | |
| --- | --- | --- | --- |
| Preferred Term | Number of reports | Preferred Term | Number of reports |
| Nausea | 1,470 | Arrhythmia | 94 |
| Dizziness | 1,416 | Intestinal obstruction | 82 |
| Headache | 1,124 | Erectile dysfunction | 79 |
| Fatigue | 1,114 | Suicidal ideation | 64 |
| Diarrhoea | 1015 | Circulatory collapse | 44 |
| Pruritus | 601 | Syncope | 43 |
| Vomiting | 479 | Hallucination | 40 |
| Rash | 478 | Angina pectoris | 34 |
| Weight increased | 477 | Haemorrhage | 33 |
| Hyperhidrosis | 443 | Haematochezia | 32 |
| Myalgia | 443 | Angioedema | 22 |
| Palpitations | 372 | Deafness | 22 |
| Dyspnoea | 371 | Anaphylactic shock | 20 |
| Somnolence | 363 | Polyneuropathy | 18 |
| Abdominal pain | 339 | Peripheral neuropathy | 17 |
| Arthralgia | 324 | Thrombosis | 16 |
| Abdominal pain upper | 313 | Paralysis | 15 |
| Cough | 293 | Loss of consciousness | 15 |
| Sleep disorder | 252 | Seizure | 14 |
| Rash pruritic | 251 | Cardiac flutter | 13 |

E-table 4: Number of ADRs for suspected compounds marketed since 1/1/2015 (top 20)

| Compound | ATC Code | Number of ADRs (%) |
| --- | --- | --- |
| Edoxaban | B01AF03 | 140 (18.9) |
| Evolocumab | C10AX13 | 95 (12.9) |
| zoster, purified antigen | J07BK03 | 86 (11.6) |
| Dulaglutide | A10BJ05 | 73 (9.9) |
| valsartan and sacubitril | C09DX04 | 50 (6.8) |
| Erenumab | N02CD01 | 31 (4.2) |
| Ixekizumab | L04AC13 | 24 (3.2) |
| Vortioxetine | N06AX26 | 21 (2.8) |
| Baricitinib | L04AA37 | 19 (2.6) |
| Tofacitinib | L04AA29 | 18 (2.4) |
| emtricitabine, tenofovir alafenamide and bictegravir | J05AR20 | 16 (2.2) |
| Ivermectin | D11AX22 | 16 (2.2) |
| Ocrelizumab | L04AA36 | 16 (2.2) |
| Olaparib | L01XX46 | 14 (1.9) |
| Alirocumab | C10AX14 | 13 (1.8) |
| house dust mites | V01AA03 | 13 (1.8) |
| Secukinumab | L04AC10 | 13 (1.8) |
| Brivaracetam | N03AX23 | 12 (1.6) |
| papillomavirus (human types 6, 11, 16, 18, 31, 33, 45, 52, 58) | J07BM03 | 10 (1.4) |
| sofosbuvir and velpatasvir* | J05AP55 | 9 (1.2) |
| benralizumab* | R03DX10 | 9 (1.2) |

*Due to the same number, both compounds are shown

E-table 5: Most frequently reported ADRs (number of reports, top ten, Preferred Terms according to MedDRA) related to the three most frequently suspected compounds marketed since 1/1/2015.

| Adverse drug reaction | Number of reports | Suspected compound (top three) | ATC Code | Number of reports |
| --- | --- | --- | --- | --- |
| Nausea | 34 | dulaglutide | A10BJ05 | 13 |
|  |  | valsartan and sacubitril | C09DX04 | 5 |
|  |  | edoxaban | B01AF03 | 4 |
| Fatigue | 34 | zoster, purified antigen | J07BK03 | 5 |
|  |  | valsartan and sacubitril | C09DX04 | 4 |
|  |  | evolocumab | C10AX13 | 4 |
|  |  | vortioxetine | N06AX26 | 4 |
| Headache | 30 | zoster, purified antigen | J07BK03 | 9 |
|  |  | dulaglutide | A10BJ05 | 3 |
|  |  | evolocumab | C10AX13 | 3 |
| Dizziness | 29 | edoxaban | B01AF03 | 10 |
|  |  | dulaglutide | A10BJ05 | 6 |
|  |  | valsartan and sacubitril | C09DX04 | 5 |
| Pruritus | 20 | edoxaban | B01AF03 | 8 |
|  |  | evolocumab | C10AX13 | 4 |
|  |  | zoster, purified antigen | J07BK03 | 2 |
|  |  | erenumab | N02CD01 | 2 |
| Pyrexia | 16 | zoster, purified antigen | J07BK03 | 11 |
|  |  | evolocumab | C10AX13 | 2 |
| Arthralgia | 15 | evolocumab | C10AX13 | 8 |
|  |  | zoster, purified antigen | J07BK03 | 2 |
| Myalgia | 15 | evolocumab | C10AX13 | 7 |
|  |  | zoster, purified antigen | J07BK03 | 4 |
|  |  | benralizumab | R03DX10 | 2 |
| Dyspnoea | 12 | edoxaban | B01AF03 | 5 |
|  |  | ixekizumab | L04AC13 | 2 |
| Rash | 11 | edoxaban | B01AF03 | 4 |
|  |  | valsartan and sacubitril | C09DX04 | 2 |
